# Supplementary material for: Crowdsource authoring as a tool for enhancing the quality of competency assessments in healthcare professions
Source: PLoS One. 2023 Nov 2;18(11):e0278571. doi: 10.1371/journal.pone.0278571 (PMC10621860; doi:10.1371/journal.pone.0278571)
Supplement: S1 Fig — (DOCX) [file pone.0278571.s001.docx]

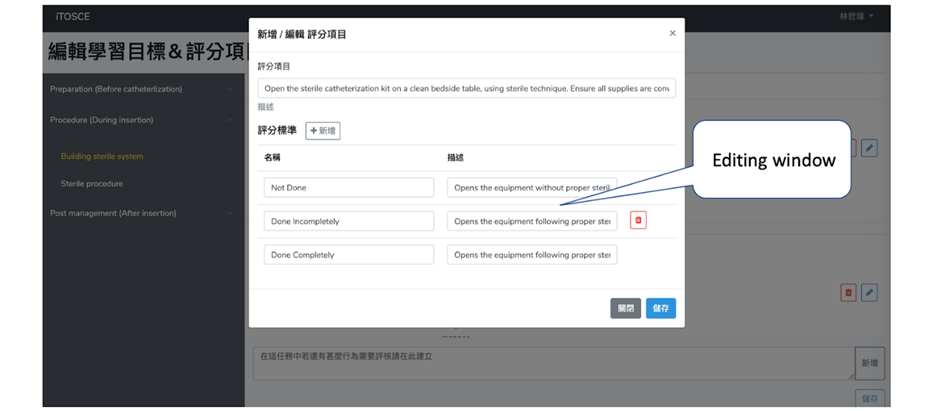


**S1 Fig.** **The CAAT version 1.0 editing window.** Here, assessments, results and data can be inputted, edited, and stored by users according to their needs and what they are experiencing.
