# Supplementary figures and images for: Crowdsource authoring as a tool for enhancing the quality of competency assessments in healthcare professions
Source: PLoS One. 2023 Nov 2;18(11):e0278571. doi: 10.1371/journal.pone.0278571 (PMC10621860; doi:10.1371/journal.pone.0278571)

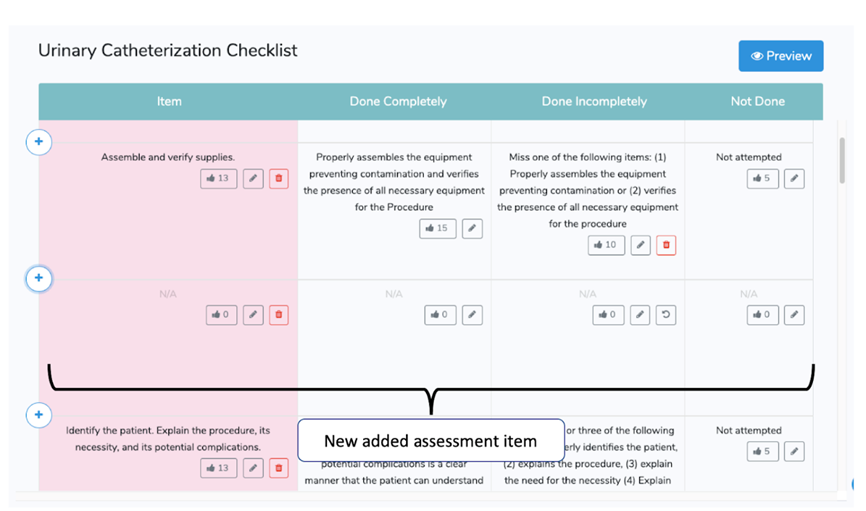


**S2 Fig. The CAAT display of a newly added assessment item.**

Supplement: S2 Fig — (DOCX) [file pone.0278571.s002.docx]

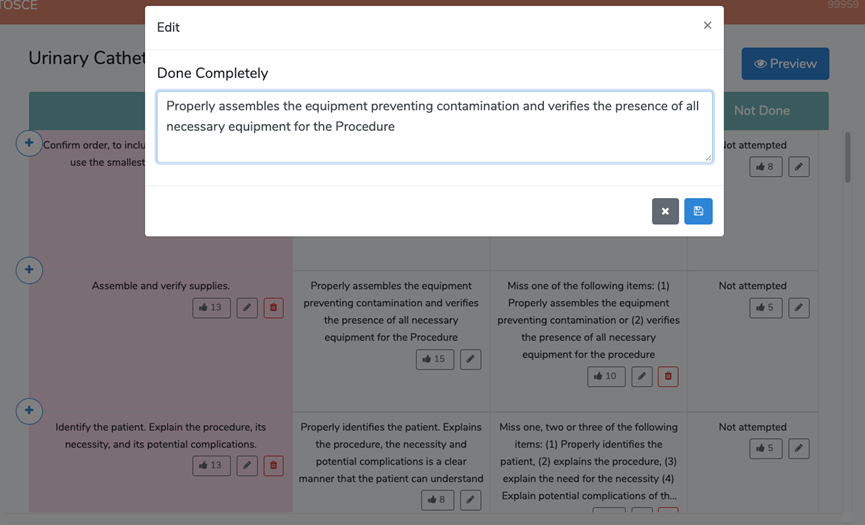


**S3 Fig**. **The displaying of a completed asessment on the CAAT system.**

Supplement: S3 Fig — (DOCX) [file pone.0278571.s003.docx]

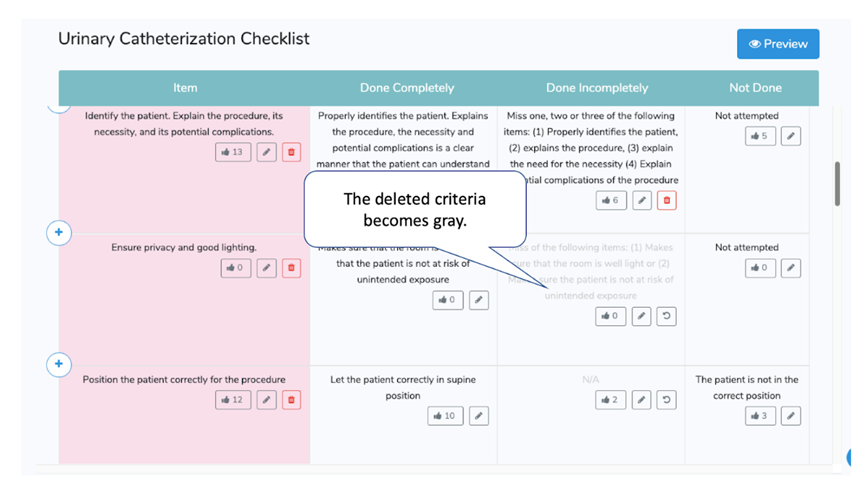


**S4 Fig 4**. **The CAAT interface displaying a deleted assessment criteria**

Supplement: S4 Fig — (DOCX) [file pone.0278571.s004.docx]
