# Supplementary material for: Crowdsource authoring as a tool for enhancing the quality of competency assessments in healthcare professions
Source: PLoS One. 2023 Nov 2;18(11):e0278571. doi: 10.1371/journal.pone.0278571 (PMC10621860; doi:10.1371/journal.pone.0278571)
Supplement: S5 Fig — (DOCX) [file pone.0278571.s005.docx]

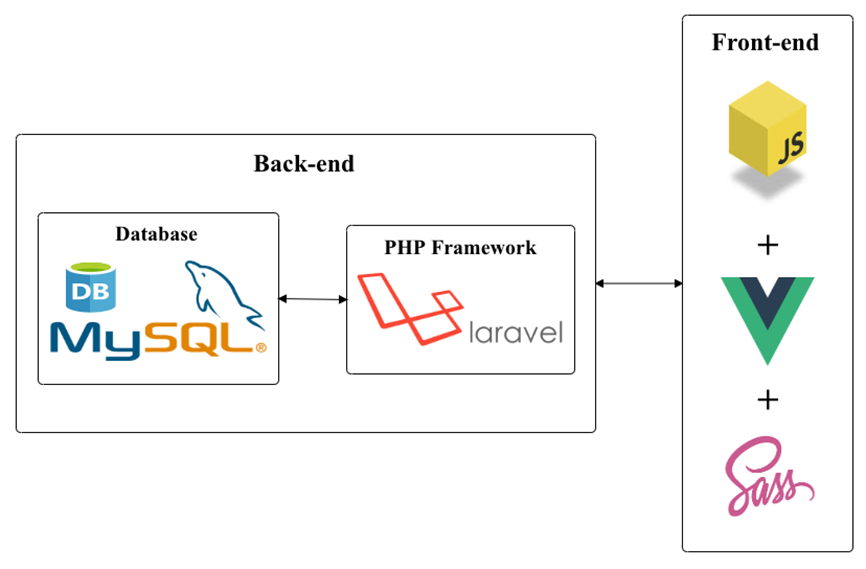


**S5 Fig**. CAAT’s Information system framework.

In addition to details of figures in out manuscript and due to lack of space thereof, we would like to provide some details of the CAAT’s information system. The main framework for the content management system of CAAT is Laravel, a web application framework, expressive, and with elegant syntax for reducing web development difficulties through various easy and common tasks that are used on the market. In constructing Laravel, this study uses Hypertext Preprocessor (PHP) as a programming language. The database uses MySQL because it is an open source with high efficiency, low cost, reliability, and well-known. Front-end of the CAAT uses Vue.js for the main framework and the JavaScript programming language. Vue.js supports JavaScript to build a user interaction framework, and it can also be applied to building a single Web page framework with a concise and structured way to develop Web frameworks. Moreover, Vue.js focuses on the view layout in the model–view–controller (MVC). Vue.js can easily carry out interactions between the viewer and the program model through different subjects. Another strength of Vue.js is that it can easily obtain and update data. The layout of the front end for CAAT uses Bootstrap and sass. The figure below displays the framework of the information system for the CAAT.
