# Supplementary material for: Crowdsource authoring as a tool for enhancing the quality of competency assessments in healthcare professions
Source: PLoS One. 2023 Nov 2;18(11):e0278571. doi: 10.1371/journal.pone.0278571 (PMC10621860; doi:10.1371/journal.pone.0278571)
Supplement: S1 Table — (DOCX) [file pone.0278571.s006.docx]

**S1 Table**. **Correlations between experience and the CAAT acceptance**

|  | Teaching | Using | Developing | Time | Time |
| --- | --- | --- | --- | --- | --- |
| Item | Experience | Checklist | Checklist | (traditional) | (CAAT) |
| 01 | .246^+^ | .142 | .181 | -.011 | .042 |
| 02 | .320* | .180 | .218 | -.017 | .007 |
| 03 | -.210 | -.097 | -.119 | -.003 | .066 |
| 04 | -.193 | -.200 | -.179 | .118 | .156 |
| 05 | -.252^+^ | -.117 | -.125 | -.015 | .019 |
| 06 | .138 | .158 | .155 | -.033 | -.041 |
| 07 | -.057 | .169 | .149 | -.035 | -.010 |
| 08 | -.145 | -.139 | -.147 | .003 | .025 |
| 09 | -.212 | -.283* | -.266^+^ | .156 | .163 |
| 10 | -.262^+^ | -.257^+^ | -.252^+^ | .164 | .178 |
| 11 | -.198 | -.079 | -.076 | .036 | .062 |
| 12 | -.192 | -.193 | -.208 | .191 | .193 |
| 13 | .272^+^ | .099 | .079 | .141 | .162 |
| 14 | -.037 | -.259^+^ | -.202 | .157 | .174 |

Pearson correlation; ^+^ *p* < .10; * *p* < .05.

To know more specific details about users’ experiences and acceptance of the CAAT system we performed a correlation analysis among users’ relevant experience and CAAT acceptance to see if the former can influence the latter in any way or form. Regarding the correlation between relevant experiences and CAAT acceptance, there were only two results reaching statistical significance, and the other eight results showed marginal significance. According to the data obtained the most meaningful were the correlations between teaching experience and CAAT acceptance. The results demonstrated that those having more teaching experience had a higher score: (ii) they can decide easily which item to use for checking students’ performance via CAAT for urinary catheter insertion (*r* = .320, *p* < .05). Teaching experience was also positively correlated to the other two items, having only marginal significance. The results showed higher agreement for “(i) I can decide quickly which item the experts want to use for checking students’ performance during urinary catheter insertion” from those having more teaching experience (*r* = .246, *p* < .10). In another situation, the result showed higher agreement for “(xiii) I intend to revisit the CAAT in the future” from those having more teaching experience (*r* = .272, *p* < .10). Interestingly, there were another two items negatively correlated to teaching experience. The result showed a lower correlation for “(v) the interaction with CAAT is clear and understandable” from those having more teaching experience (*r* = -.252, *p* < .10). Furthermore, results showed a lower correlation for “(x) CAAT is exciting to use” from those having more teaching experience (*r* = -.262, *p* < .10).

Another important aspect of the CAAT acceptance is the experience of using a checklist for educational purposes. However, those correlations reaching statistical significance or marginal significance were negative. For instance, the results demonstrated that those having more experience using a checklist for educational purposes had a lower score for “(ix) the CAAT is enjoyable (*r* = -.283, *p* < .05)”. Moreover, the results also reflected a lower correlation for “(x) the CAAT is unappealing (*r* = -.257, *p* < .10)” and “(xiv) I will use the CAAT next time I need to generate OSCE checklist (*r* = -.259, *p* < .10)” from those having more experience using a checklist for educational purpose.
