# Supplementary material for: Crowdsource authoring as a tool for enhancing the quality of competency assessments in healthcare professions
Source: PLoS One. 2023 Nov 2;18(11):e0278571. doi: 10.1371/journal.pone.0278571 (PMC10621860; doi:10.1371/journal.pone.0278571)
Supplement: S2 Table — (DOCX) [file pone.0278571.s007.docx]

**S2 Table. Differences in the CAAT acceptance between senior and junior experts.**

|  | **Teaching experience ^a^** | | **Using checklist** | | **Developing checklist** | |
| --- | --- | --- | --- | --- | --- | --- |
| **Item** | MD | *T* | MD | *t* | MD | *t* |
| **01** | 0.02 | 0.075 | 0.03 | 0.092 | -0.20 | -0.720 |
| **02** | 0.16 | 0.665 | 0.08 | 0.318 | 0.00 | 0.000 |
| **03** | -0.19 | -0.700 | -0.34 | -1.232 | -0.24 | -0.897 |
| **04** | -0.55 | -1.527 | -0.53 | -1.506 | -0.48 | -1.472 |
| **05** | -0.51* | -2.083 | -0.11 | -0.419 | -0.28 | -1.115 |
| **06** | -0.13 | -0.506 | 0.09 | 0.360 | -0.04 | -0.154 |
| **07** | -0.19 | -0.914 | 0.12 | 0.567 | 0.00 | 0.000 |
| **08** | -0.36 | -1.303 | -0.36 | -1.226 | -0.44 | -1.615 |
| **09** | -0.61* | -2.410 | -0.69** | -2.759 | -0.44+ | -1.782 |
| **10** | -0.76* | -2.578 | -0.57+ | -1.926 | -0.60* | -2.021 |
| **11** | -0.48 | -1.638 | -0.29 | -1.009 | -0.32 | -1.172 |
| **12** | -0.49 | -1.648 | -0.55+ | -1.872 | -0.48+ | -1.729 |
| **13** | 0.11 | 0.372 | -0.06 | -0.200 | -0.16 | -0.558 |
| **14** | -0.53 | -1.539 | -0.68* | -2.007 | -0.72* | -2.158 |
| **Useful** | -0.14 | -0.598 | -0.19 | -0.837 | -0.23 | -1.020 |
| **Easy** | -0.30 | -1.454 | -0.06 | -0.301 | -0.19 | -0.918 |
| **Enjoy** | -0.58* | -2.194 | -0.52+ | -1.986 | -0.46 | -1.831 |
| **BI** | -0.21 | -0.728 | -0.37 | -1.295 | -0.44 | -1.563 |

Independent t-test; ^+^ *p* < .10; * *p* < .05; ** *p* < .01; ^a^ senior (more than 8 years teaching experience, n = 21) and junior (within 8 years teaching experience, n = 29); ^b^ senior (experience on using checklist for educational purpose more than 6 years, n = 22) and junior (experience on using a checklist for educational purpose within 6 years, n = 28); ^c^ senior (experience on developing checklist more than 4 years, n = 25) and junior (experience on developing checklist within 4 years, n = 25); BI, behavior intention; MD., mean difference.

There were some interesting findings when this study investigated the differences in the CAAT acceptance between senior experts and junior experts (Supplementary Table 2). According to teaching experience, senior experts had significantly lower scores than junior experts for “(v) the interaction with CAAT is clear and understandable (MD = -0.51, *t* = -2.08, *p* < .05); (ix) the CAAT is enjoyable (MD = -0.61, *t* = -2.41, *p* < .05), and (x) the CAAT is exciting (MD = -0.76, *t* = -2.58, *p* < .05)”. According to the experience in using a checklist for educational purpose, senior experts had significantly lower scores than junior experts for the following items: (ix) the CAAT is enjoyable (MD = -0.69, *t* = -2.76, *p* < .01) and (xiv) I will use the CAAT next time I need to generate OSCE checklist (MD = -0.68, *t* = -2.01, *p* < .05)”. Additionally, according to the experience for developing a checklist, senior experts had significantly lower scores than junior experts for “(x) the CAAT is exciting (MD = -0.60, *t* = -2.02, *p* < .05) and (xiv) I will use the CAAT next time I need to generate an OSCE checklist (MD = -0.72, *t* = -2.16, *p* < .05)”. However, experts with different experiences in teaching, using checklist for educational purpose, and developing checklist showed insignificant results for usefulness, ease of use, and behavior intention. There was only one significant finding showing that experts with more teaching experience felt lower enjoyment than those with less teaching experience when using the CAAT (MD = -0.58, *t* = -2.19, *p* < .05).
