# Supplementary material for: Crowdsource authoring as a tool for enhancing the quality of competency assessments in healthcare professions
Source: PLoS One. 2023 Nov 2;18(11):e0278571. doi: 10.1371/journal.pone.0278571 (PMC10621860; doi:10.1371/journal.pone.0278571)
Supplement: Attachment — Submitted filename: PONE-D-21-35425_reviewer.pdf [file pone.0278571.s009.pdf]

PLOS ONE

# Crowdsource Authoring as a Tool for Enhancing the Quality of Competency Assessments in Healthcare Professions

--Manuscript Draft--

| | |
|---|---|
| **Manuscript Number:** | PONE-D-21-35425 |
| **Article Type:** | Research Article |
| **Full Title:** | Crowdsource Authoring as a Tool for Enhancing the Quality of Competency Assessments in Healthcare Professions |
| **Short Title:** | Crowdsource Authoring Can Enhance Healthcare Competency Assessments |
| **Corresponding Author:** | Yu-Chuan (Jack) Li<br>Taipei Medical University<br>Taipei, TAIWAN |
| **Keywords:** | CAAT, healthcare competence, OSCE, technology acceptance model |
| **Abstract:** | The current Objective Structured Clinical Examination (OSCE) is complex, costly, and difficult to provide high-quality assessments. The Crowdsource Authoring Assessment Tool (CAAT) allows the creation and sharing of assessment tools easily by editing and customizing them to match specific needs and provide higher quality. Competency assessment international experts (n = 50) were asked to 1) participate in the CAAT system beta test, 2) edit a urinary catheterization checklist using CAAT, and 3) complete a Technology Acceptance Model (TAM) questionnaire consisting of 14 items to evaluate its four domains. The median time for developing a new checklist using the CAAT was 65.76 minutes whereas the traditional method required 167.90 minutes. The CAAT system enabled quicker checklist creation and editing regardless of the experience and native language of participants. Participants also expressed the CAAT enhanced checklist development with 96% of them willing to recommend this tool to others. The use of a crowdsource authoring tool as revealed by this study has efficiently reduced the time to almost a third it would take when using the traditional method. In addition, it allows collaborations to partake on a simple platform which also promotes contributions in checklist creation, editing, and rating. |
| **Order of Authors:** | Che-Wei Lin, MD, PhD |
| | Daniel L Clinciu, PhD, MSc |
| | Daniel Salcedo, MD, MSc |
| | Chih-Wei Huang, PhD |
| | Yi No Kang |
| | Yu-Chuan (Jack) Li |
| **Additional Information:** | |
| **Question** | **Response** |
| **Financial Disclosure**<br><br>Enter a financial disclosure statement that describes the sources of funding for the work included in this submission. Review the submission guidelines for detailed requirements. View published research articles from *PLOS ONE* for specific examples.<br><br>This statement is required for submission and **will appear in the published article** if | This is an unfunded study |

Powered by Editorial Manager® and ProduXion Manager® from Aries Systems Corporation

| | |
|---|---|
| the submission is accepted. Please make sure it is accurate.<br><br>**Unfunded studies**<br>Enter: *The author(s) received no specific funding for this work.*<br><br>**Funded studies**<br>Enter a statement with the following details:<br>• Initials of the authors who received each award<br>• Grant numbers awarded to each author<br>• The full name of each funder<br>• URL of each funder website<br>• Did the sponsors or funders play any role in the study design, data collection and analysis, decision to publish, or preparation of the manuscript?<br>• **NO** - Include this sentence at the end of your statement: *The funders had no role in study design, data collection and analysis, decision to publish, or preparation of the manuscript.*<br>• **YES** - Specify the role(s) played.<br><br>* typeset | |
| **Competing Interests**<br><br>Use the instructions below to enter a competing interest statement for this submission. On behalf of all authors, disclose any competing interests that could be perceived to bias this work—acknowledging all financial support and any other relevant financial or non-financial competing interests.<br><br>This statement is required for submission and **will appear in the published article** if the submission is accepted. Please make sure it is accurate and that any funding sources listed in your Funding Information later in the submission form are also declared in your Financial Disclosure statement.<br><br>View published research articles from *PLOS ONE* for specific examples. | Authors declare no competing interests |

Powered by Editorial Manager® and ProduXion Manager® from Aries Systems Corporation

| | |
|---|---|
| **NO authors have competing interests**<br><br>Enter: *The authors have declared that no competing interests exist.*<br><br>**Authors with competing interests**<br><br>Enter competing interest details beginning with this statement:<br><br>*I have read the journal's policy and the authors of this manuscript have the following competing interests: [insert competing interests here]*<br><br>* typeset | |
| **Ethics Statement**<br><br>Enter an ethics statement for this submission. This statement is required if the study involved:<br><br>• Human participants<br>• Human specimens or tissue<br>• Vertebrate animals or cephalopods<br>• Vertebrate embryos or tissues<br>• Field research<br><br>Write "N/A" if the submission does not require an ethics statement.<br><br>General guidance is provided below. Consult the submission guidelines for detailed instructions. **Make sure that all information entered here is included in the Methods section of the manuscript.** | IRB: TMU-JIRB-N201603019.<br>Taipei Medical University |

| | |
|---|---|
| **Format for specific study types**<br><br>**Human Subject Research (involving human participants and/or tissue)**<br>• Give the name of the institutional review board or ethics committee that approved the study<br>• Include the approval number and/or a statement indicating approval of this research<br>• Indicate the form of consent obtained (written/oral) or the reason that consent was not obtained (e.g. the data were analyzed anonymously)<br><br>**Animal Research (involving vertebrate animals, embryos or tissues)**<br>• Provide the name of the Institutional Animal Care and Use Committee (IACUC) or other relevant ethics board that reviewed the study protocol, and indicate whether they approved this research or granted a formal waiver of ethical approval<br>• Include an approval number if one was obtained<br>• If the study involved *non-human primates*, add *additional details* about animal welfare and steps taken to ameliorate suffering<br>• If anesthesia, euthanasia, or any kind of animal sacrifice is part of the study, include briefly which substances and/or methods were applied<br><br>**Field Research**<br><br>Include the following details if this study involves the collection of plant, animal, or other materials from a natural setting:<br>• Field permit number<br>• Name of the institution or relevant body that granted permission | |
| **Data Availability**<br><br>Authors are required to make all data underlying the findings described fully available, without restriction, and from the time of publication. PLOS allows rare exceptions to address legal and ethical concerns. See the PLOS Data Policy and FAQ for detailed information. | Yes - all data are fully available without restriction |

| | |
|---|---|
| A Data Availability Statement describing where the data can be found is required at submission. Your answers to this question constitute the Data Availability Statement and **will be published in the article**, if accepted.<br><br>**Important:** Stating 'data available on request from the author' is not sufficient. If your data are only available upon request, select 'No' for the first question and explain your exceptional situation in the text box.<br><br>Do the authors confirm that all data underlying the findings described in their manuscript are fully available without restriction? | |
| **Describe where the data may be found in full sentences. If you are copying our sample text, replace any instances of XXX with the appropriate details.**<br><br>• If the data are **held or will be held in a public repository**, include URLs, accession numbers or DOIs. If this information will only be available after acceptance, indicate this by ticking the box below. For example: *All XXX files are available from the XXX database (accession number(s) XXX, XXX.)*.<br>• If the data are all contained **within the manuscript and/or Supporting Information files**, enter the following: *All relevant data are within the manuscript and its Supporting Information files.*<br>• If neither of these applies but you are able to provide **details of access elsewhere**, with or without limitations, please do so. For example:<br><br>*Data cannot be shared publicly because of [XXX]. Data are available from the XXX Institutional Data Access / Ethics Committee (contact via XXX) for researchers who meet the criteria for access to confidential data.*<br><br>*The data underlying the results presented in the study are available from (include the name of the third party* | Additional data if needed are available from the Taipei Medical University Institutional Data Access |

| | |
|---|---|
| *and contact information or URL).*<br>• This text is appropriate if the data are owned by a third party and authors do not have permission to share the data.<br><br>* typeset | |
| Additional data availability information: | Tick here if the URLs/accession numbers/DOIs will be available only after acceptance of the manuscript for publication so that we can ensure their inclusion before publication. |

Manuscript
Click here to access/download;Manuscript;Crowdsource Authoring as a Tool_cmpb.docx

**Title Page**

# Crowdsource Authoring as a Tool for Enhancing the Quality of Competency Assessments in Healthcare Professions

**Che-Wei Lin, MD, PhD**
Department of Education and Humanities in Medicine
School of Medicine, College of Medicine, Taipei Medical University
Taipei, Taiwan
Email: thomas0205@gmail.com

**Daniel L. Clinciu, PhD, MSc**
Graduate Institute of Biomedical Science
China Medical University
Taichung, Taiwan
Email: celdan99@gmail.com

**Daniel Salcedo, MD, MHPE**
Taipei Municipal Wanfang Hospital
Taipei, Taiwan
Email: dsalcedo@tmu.edu.tw

**Chih-Wei Huang, PhD**
International Center for Health Information Technology
College of Medical Science and Technology
Taipei Medical University, Taipei, Taiwan
Email: gracehuang@tmu.edu.tw

**Yi No Kang, MSc**
Department of Education, Taipei Medical University Hospital, Taipei, Taiwan
Evidence-Based Medicine Center, Wan Fang Hospital, Taipei Medical University
Taipei, Taiwan

Email: kynacad@gmail.com

**Yu-Chuan(Jack) Li**, MD, PhD, Professor (Correspondent)
Graduate Institute of Biomedical Informatics, College of Medical Science and Technology, Taipei Medical University, Taipei, Taiwan
International Center for Health Information Technology (ICHIT), Taipei Medical University, Taipei, Taiwan
Research Center of Big Data and Meta-analysis, Wan Fang Hospital, Taipei Medical University, Taipei, Taiwan
Department of Dermatology, Wan Fang Hospital, Taipei, Taiwan
TMU Research Center of Cancer Translational Medicine, Taipei Medical University, Taiwan
Email: jaak88@gmail.com
Tel: +886-2-27361661#3111~3114

**Abstract**

The current Objective Structured Clinical Examination (OSCE) is complex, costly, and difficult to provide high-quality assessments. The Crowdsource Authoring Assessment Tool (CAAT) allows the creation and sharing of assessment tools easily by editing and customizing them to match specific needs and provide higher quality. Competency assessment international experts (n = 50) were asked to 1) participate in the CAAT system beta test, 2) edit a urinary catheterization checklist using CAAT, and 3) complete a Technology Acceptance Model (TAM) questionnaire consisting of 14 items to evaluate its four domains. The median time for developing a new checklist using the CAAT was 65.76 minutes whereas the traditional method required 167.90 minutes. The CAAT system enabled quicker checklist creation and editing regardless of the experience and native language of participants. Participants also expressed the CAAT enhanced checklist development with 96% of them willing to recommend this tool to others. The use of a crowdsource authoring tool as revealed by this study has efficiently reduced the time to almost a third it would take when using the traditional method. In addition, it allows collaborations to partake on a simple platform which also promotes contributions in checklist creation, editing, and rating.

**Keywords**: CAAT, healthcare competence, OSCE, technology acceptance model

## 1. Introduction

During the past 5 decades, the focus of the training of medical has shifted dramatically towards competency-based medical education (CBME) [1]. Thus, the development of new assessment methodologies for the accurate measurement of competency in an accurate and unbiased form was needed [2]. The traditional Objective Structured Clinical Examination (OSCE) was developed in the 1970s as a replacement for the Clinical Examination, which was a required practical competency exam for graduating physicians in the United Kingdom [3]. This examination is a 1-hour encounter with a patient, where candidates are requested to obtain a clinical history and conduct a physical examination. After the allotted time, the candidate would then meet with a group of examiners to discuss the case further, approximately 20 minutes. Then, the candidate is required to perform a focused examination of several additional patients and discuss the clinical findings with the examiners.

OSCE has been in place for several decades, however, it attracted a significant amount of criticism, due to lack of reliability in measuring candidates' clinical competence and the effect of no implemented standardization of cases and examiners. Later developments to improve the quality of OSCE in terms of validity and reliability scores were the design of scenario and assessment tool design checklists, more frequently used as interactive digital files [4]. The basic procedure for developing OSCE checklists includes three main stages for developing assessment tools to measure clinical skills [5]. These stages constitute the traditional method of checklist development which is still used by numerous institutions around the world: 1) Preliminary List of Measurable Steps - the OSCE station authors develop an initial list of all key and measurable steps in the clinical skill to be evaluated in a particular station; 2) Specialist Assessment - experts with particular skills assess the OSCE station's draft checklists and provide feedback and

suggestions for improvement; 3) Field Testing - a mock examination using realistic OSCE conditions.

Developing a new OSCE station checklist is complex, time-consuming, requires significant resources and participation of experts, mock examiners, and candidates [6-9]. However, the electronic checklist system, a worldwide recent development, can eliminate missing data and decrease post-assessment working loads. Therefore, the quality of future medical education will rely on increased collaboration between institutions providing medical programs [10-12]. These inter-institutional collaborations could enhance the quality of medicine using joint development of educational programs and activities by combining the resources and capabilities of different medical schools [13]. One example is the MedEdPORTAL system (Figure 1). It recognizes author contributions by displaying publication metrics on its website. The built-in credit system displays specific metrics such as the number of views, number of downloads, and the conversion rate from views to downloads. The system also tracks social media coverage and displays an “attention score” calculated based on these metrics. The system tracks the impact of the shared resource but indirectly acts as a reward for the authors since it is an indicator of quality and relevance.

One of the greatest examples of successful crowd authoring and crowdsourcing not only in the medical field but all other fields is Wikipedia. This is an online encyclopedia that is completely crowd authored; anyone with knowledge of a particular subject can contribute. Contributions are added to the database after a peer-review process to ensure accuracy. By December 2020, Wikipedia contained more than 6 million crowdsourced articles and has become one of the largest repositories of online information in the world [14,15]. Crowdsourcing comes from a less-specific, more public group and its advantages may include improved costs, speed,

quality, flexibility, scalability, or diversity. Therefore, this study aims to build a Crowdsource Assessment Authoring Tool (CAAT) which can generate an OSCE checklist easier and more efficiently.

## 2. Methods

The online OSCE generator checklist CAAT conceptualized in this study was comprised of three phases. The first phase establishes frameworks for procedure-based OSCE checklists and online platforms (Figure 2). The second phase is aimed at detecting and fixing bugs (e.g. problems with software, programs). The third phase investigates the user's acceptance of the CAAT. The first two phases are designed for practical purposes, and the third phase contains the theoretical investigation.

### *2.1. Consent and ethics statement*

Participants were given informed consent about the study and were explained in detail how their participation can help improve collaboration, checklist creation and to save time and resources in the healthcare profession. This study was approved by the Ethics Committee Review Board at Taipei Medical University at Taipei Medical University Hospital. IRB: TMU-JIRB-N201603019.

### *2.2. Participants in the development of the CAAT framework*

1. Medical doctors and educational scholars

2. Medical doctors must have at least three years of experience participating in the national OSCE certification or developing OSCE checklists.

3. Educational scholars must have at least three years of experience handling medical education projects or have published three medical education studies in SCI journals.

Three medical doctors and three educational scholars fulfilling the above criteria participated in the study with a total of 6 panels in the focus group meetings (minimum requirement for the number of panels in the group meeting n=5 for the consensus group of different disciplines).

*2.3. Phase I: Development of the framework for the CAAT*

Researchers examined the recent version of the national OSCE for drafting a CAAT framework. During the drafting process, they followed the concept of ontology or mind map in order to identify the elements of the OSCE checklist and to identify their relationships. As clinical skills completion occurs in a step-by-step manner, an important element of OSCE is time series. Thus, the first layer for the CAAT (time) consists of preparation, processing, post-management. The second layer is “the key tasks” for each clinical skill. Then, researchers drafted the first focus group framework.

During the focus group meeting, researchers presented the primary concept and framework of the CAAT and guided the group in developing an OSCE checklist. According to the experience exchange and verified the researchers further modified and enhanced the elements used in developing the OSCE checklist. Afterward, the researchers combined their knowledge and skills to extract the concepts/elements for developing an OSCE checklist. This cycle continued until no new information or modifications were produced. Then, researchers turned their confirmed findings to the engineer which implemented the CAAT’s IT framework (alpha 1.0 version, Figure 3).

*2.4. Phase II: CAAT problems detection and fixing*

After the engineer built the first version of the CAAT (alpha 1.0), the researchers examined the systematic problems with other invited experts. Then, the study called the second focus group to identify and fix existing problems. The same criteria for expert panels were used as in Phase I: 1) medical doctors, 2) educational scholars and 3) their required qualifications.

Five experts tested the Phase II of CAAT in a limited period (one week). In this phase, the researchers addressed the gap between IT design and user's needs and identified some system problems. The engineer re-designed and adjusted the CAAT according to the recommendations of the panels, and the researchers re-tested the modified CAAT (alpha 1.1 version) using various medical faculty. The adjusting cycle continued until no further suggestions were given and no problems or issues were encountered and the CAAT (beta 1.0 version, Figure 3) was approved for phase III test and survey.

*2.5. Phase III: The CAAT (beta version) for user test and survey*

To further verify the CAAT (beta 1.0 version) can benefit Taiwan and healthcare systems worldwide, a user test and survey for the OSCE checklist development were implemented. The initial sample size quota for this particular survey was 50 experts. Therefore, the study invited 60 experts from 9 different countries within the three affiliated international simulation centers to participate in Phase III development. The invited experts, faculty members, and/or researchers all attended international assessment training courses between 2010 to 2019.

The CAAT user experience survey was performed through Google docs and included background information, the CAAT acceptance scale, and the CAAT impact. To obtain unbiased feedback, this study used an anonymous survey format (See appendix). The CAAT acceptance scale was adopted from a verified scale [16], and consisted of 14 items reflecting four domains

of a technology acceptance model. The domains are perceived in terms of user-friendliness, usefulness, attractiveness, and intention of use. The scale for information system user acceptance was verified and the four domains revealed the following: The Cronbach's alpha for the usefulness domain was .90, the alpha for user friendly was .87, the alpha for attractiveness was .86, and the alpha for the behavioral intention was .87 (Table 2).

The CAAT acceptance scale involved four additional domains. The usefulness domain consists of items 1 to 4, the user-friendliness consists of items 5 to 8, attractiveness consists of items 9 to 12, and the behavioral intention consists of item 13 to item 14. Aside from the attractiveness domain, the other three domains used a 7-point Likert scale. The attractiveness domain used a 7-point bipolar scale.

*2.6. Statistical analysis*

This study used descriptive statistics (percentage event and mean with SD) for background information and inferential statistics for testing the study's hypothesis. The percentage event represented nationality, gender, and occupation. The SD mean represented experience for clinical skill teaching, checklists used for education purposes, checklist development, and the average time for developing a checklist.

For inferential statistics, an independent sample t-test, Pearson correlation, one-sample t-test, and dependent sample t-test were conducted as per hypotheses. Pearson correlation served the analysis of the correlation between the user acceptance of the CAAT and experience on clinical skill teaching (hypothesis 1), experience for checklists used in educational purposes, and experience for checklist development (hypothesis 2). A one-sample t-test was used for testing the acceptance of the CAAT among the experts (hypothesis 3). Since the questionnaire was based on

a 7-point scale, the test value for the one-sample t-test was set at 5. The dependent sample t-test assessed the difference in time for developing a checklist between the CAAT and the traditional OSCE (hypothesis 4). As the p-value was lower than 0.05, the result reached statistical significance.

## 3. Results

From the 60 surveys collected 50 were included in this study; 10 removed due to incomplete data. The 50 experts were from China (n = 19), Hong Kong (n = 1), Japan (n = 1), Malaysia (n = 4), Singapore (n = 1), South Korea (n = 2), Taiwan (n = 10), Thailand (n = 3), and USA (n = 9). Thirty-four (68%) were males, and 16 (32%) were females with the following professions: physicians (n = 41, 82%), and nine administrators (n = 2), bioengineers (n = 1), operation room directors (n = 1), educators (n = 2), nurses (n = 2), occupational therapists (n = 1). The average teaching experience – 8 years; using checklist for educational purposes – 6 years; and developing a checklist – 4.5 years. The participants averaged time 90 minutes developing a new checklist through the traditional method; they only spent about 20 minutes developing a new checklist by using CAAT (Table 1).

### *3.1. Overall results of the CAAT acceptance survey*

The following feedback was obtained from the experts participating in CAAT development and testing (Tables 2 and 3): (i) I can quickly decide the items I want to use for checking students' performance in urinary catheter insertion (M = 5.94, SD = 0.98); (ii) I can easily decide the items I want to use for checking students' performance in urinary catheter insertion (M = 6.00, SD = 0.86); (iii) I can develop an accurate checklist to assess students' performance in urinary catheter insertion (M = 5.92, SD = 0.94); (iv) the CAAT provides me with information that allows me to develop an effective checklist for urinary catheter insertion (M = 5.84, SD = 1.17); (v) the

interaction with CAAT is clear and understandable (M = 6.06, SD = 0.89); (vi) the interaction with CAAT does not increase the workload when creating a checklist (M = 6.22, SD = 0.91); (vii) I find CAAT easy to use (M = 6.16, SD = 0.74); (viii) it is easy to get CAAT to do what I need (M = 6.02, SD = 0.98); (ix) I feel CAAT is enjoyable/awful (M = 6.02, SD = 0.89); (x) I feel CAAT is exciting/dull (M = 5.82, SD = 1.08); (xi) I feel CAAT is pleasant/unpleasant (M = 5.80, SD = 0.97); (xii) I feel CAAT is interesting/boring (M = 5.76, SD = 1.00); (xiii) I intend to revisit CAAT in the future (M = 6.08, SD = 1.01); and (xiv) I will use CAAT next time I need to generate OSCE checklist (M = 5.88, SD = 1.22).

### *3.2. Participants' acceptance of CAAT*

The results of the one-sample t-test with a test value of 5 showed that the experts agreed CAAT is a useful, easy, and interesting system for generating a checklist for educational purpose (Table 2). More specifically, the results showed that: (i) a user can quickly decide which items to use for checking students' performance in urinary catheter insertion (MD = 0.94, $t$ = 6.80, $p$ < .001); (ii) can easily decide which item to use for checking students' performance in urinary catheter insertion (MD = 1.00, $t$ = 8.25, $p$ < .001); (iii) can develop an accurate checklist to assess students' performance in urinary catheter insertion (MD = 0.92, $t$ = 6.89, $p$ < .001); (iv) CAAT provides information in ways to effectively develop a checklist for urinary catheter insertion (MD = 0.84, $t$ = 5.09, $p$ < .001); (v) interaction with CAAT is efficient and responsive (MD = 1.06, $t$ = 8.42, $p$ < .001); (vi) CAAT does not increase workload when creating a checklist (MD = 1.22, $t$ = 9.48, $p$ < .001); (vii) CAAT is easy to use (MD = 1.16, $t$ = 11.12, $p$ < .001); (viii) CAAT is adjusted to user's needs (MD = 1.02, $t$ = 7.37, $p$ < .001); (ix) the degree of like/dislike (MD = 1.02, $t$ = 8.09, $p$ < .001); (x) CAAT is exciting to use (MD = 0.82, $t$ = 5.36, $p$ < .001); (xi) how appealing is CAAT to user (MD = 0.80, $t$ = 5.84, $p$ < .001); (xii) how interesting is CAAT (MD = 0.76, $t$ = 5.37,

$p < .001$); (xiii) user intends to use CAAT in the future (MD = 1.08, $t = 7.58$, $p < .001$); and (xiv) user will likely use CAAT for generating the next OSCE checklist (MD = 0.88, $t = 5.09$, $p < .001$).

Overall, experts around the world that participated indicate that the CAAT is a useful (MD = 0.93, $t = 8.20$, $p < .001$), easy to use (MD = 1.12, $t = 10.79$, $p < .001$), and enjoyable (MD = 0.85, $t = 6.61$, $p < .001$) platform for generating a checklist for clinical skill. Moreover, they expressed keen interest in using the CAAT in future (MD = 0.98, $t = 6.86$, $p < .001$).

### *3.3. Effectiveness of the CAAT*

Overall, the experts used 65.76 minutes for developing a new checklist using CAAT, whereas, they spent 167.90 minutes building a new checklist traditionally (Table 3). The dependent-sample *t*-test revealed that CAAT saved 102 minutes in generating a new checklist as compared to the traditional method ($t = -2.36$, $p < .05$).

In stratified analyses, this study explored the effectiveness of the CAAT among English native speakers and non-native speakers respectively; the study also separated data for understanding the effectiveness of the CAAT among junior experts and senior experts in developing checklists. The English native speakers generated a new checklist in about 25.20 minutes using CAAT, and in 54.67 minutes traditionally. The mean difference between the two approaches was -29.47 minutes ($t = -2.98$, $p < .05$). The non-native speakers generated a new checklist using CAAT in about 83.14 minutes, and in 216.43 minutes traditionally. The mean difference between the two approaches was -133.29 minutes ($t = -2.18$, $p < .05$). When the study separated junior experts from senior experts, the results demonstrated that the junior experts generated a new checklist with CAAT in about 41.12 minutes, and with the traditional way in 96.60 minutes. The mean difference between the two approaches was -55.48 minutes ($t = -3.76$, $p = .001$). However, among the senior experts, the results demonstrated that they generated a new

checklist with CAAT in about 90.40 minutes, and with the traditional method in 239.20 minutes. The mean difference between the two approaches was -148.80 minutes with marginal significance ($t$ = -1.75, $p$ = .093). Participants/experts from around the world perceived that CAAT could change current practices of checklist development (item 12: MD = 0.76, t = 5.07, p < .001; Table 2).

## 4. Discussion

This study developed and tested the Crowdsource Authoring Assessment Tool (CAAT), a new tool and collaboration system to faster and more efficiently generate a checklist for assessing the clinical skill performance of learners. Analyzed data collected from 50 recognized international experts in the field of competency assessment revealed that CAAT can significantly reduce time and improve efficiency and ease when generating a checklist to assess a learner's clinical skills (Table 3).

The average time for participating experts was 65.76 minutes when using CAAT, whereas, they spent 167.90 minutes using OSCE, the traditional method currently used worldwide [1-3]. According to the study's results, the average time saved by using CAAT is 102.14 minutes (Table 3). In addition, Cronbach's alpha revealed high reliability for usefulness (.90), user-friendliness (.87), attractiveness (.86), and behavioral intention (.87) which are all important features of CAAT (Table 2). To analyze these important features of CAAT in an unbiased and comprehensive manner, experts from around the world were invited to participate, which are both native (30%) and non-native English speakers (70%). In addition, the experts participating in this study comprised a variety of professionals from physicians to bioengineers (Table 1). It is important to have a diverse group of experts from around the world in order to better assess the efficiency of the tool in terms of its usefulness, ease of use and from a non-English native perspective [10, 11].

The study provided certain items for participants to use to determine if they can faster and more accurately create a checklist in assessing a test taker's performance (e.g. the insertion of a urinary catheter). In the CAAT acceptance survey, value 4 is intermediate because of the 7-point scale used. Thus, a test value higher than 5 means a positive review and the items of CAAT assessed by experts received a positive acceptance overall (Table 2).

Among the limitations of our study is its small sample size. We only invited experts which participated in assessment training courses throughout the four popular simulation centers worldwide. Moreover, the experts needed to spend an additional 30 min for a real experience with CAAT prior to the survey. Even with additional announcements and promotion of CAAT, the response rate of the experts interested to participate in this study was low. The language barrier could be another limitation as two-thirds of the participants were from non-English speaking countries. The CAAT is still in its development stage and it was mostly restricted to OSCE checklists. In the future, it can be expanded to a point-of-care assessment of competency, self, and patient-based assessments.

## Declaration of conflict interest

All authors of this manuscript declare that there are no conflicting interests upon the work and submission of this manuscript.

## Acknowledgements

The authors would like to thank all the participants and all those involved in helping out with the crowdsourcing tool development and assessment.

## References

1. Harden, R., Stevenson, M., Downie, W. W., & Wilson, G. (1975). Assessment of clinical competence using objective structured examination. Br Med J, 1(5955), 447-451.
2. Howley, L. D. (2004). Performance assessment in medical education: where we've been and where we're going. Evaluation & the health professions, 27(3), 285-303.
3. Wass, V., Van der Vleuten, C., Shatzer, J., & Jones, R. (2001). Assessment of clinical competence. The Lancet, 357(9260), 945-949.
4. Hochlehnert, A., Schultz, J.-H., Möltner, A., Tımbıl, S., Brass, K., & Jünger, J. (2015). Electronic acquisition of OSCE performance using tablets. GMS Zeitschrift für Medizinische Ausbildung, 32(4).
5. OConnor, H. M., & McGraw, R. C. (1997). Clinical skills training: developing objective assessment instruments. Medical education, 31(5), 359-363. doi:DOI 10.1046/j.1365-2923.1997.d01-45.x
6. Norcini, J. J. (2003). Setting standards on educational tests. Medical education, 37(5), 464-469. doi:DOI 10.1046/j.1365-2923.2003.01495.x
7. Norman, G., Van der Vleuten, C., & De Graaff, E. (1991). Pitfalls in the pursuit of objectivity: issues of validity, efficiency and acceptability. Medical education, 25(2), 119-126.
8. 8 Daniels, V. J., & Pugh, D. (2018). Twelve tips for developing an OSCE that measures what you want. Medical teacher, 40(12), 1208-1213. doi:10.1080/0142159x.2017.1390214
9. LaRochelle, J., Durning, S. J., Boulet, J. R., van der Vleuten, C., van Merrienboer, J., & Donkers, J. (2016). Beyond standard checklist assessment: Question sequence may impact student performance. Perspectives on medical education, 5(2), 95-102.
10. Davis, D. (2018). The medical school without walls: Reflections on the future of medical education. Medical teacher, 40(10), 1004-1009.
11. Harden, R. M. (2018). Ten key features of the future medical school—not an impossible dream. *Medical teacher, 40*(10), 1010-1015.
12. Rourke, J. (2018). What does the future hold? No one knows for sure…. Medical teacher, 40(10), 980-981.
13. Ellaway, R. H., Albright, S., Smothers, V., Cameron, T., & Willett, T. (2014). Curriculum inventory: Modeling, sharing and comparing medical education programs. *Medical teacher, 36*(3), 208-215. doi:10.3109/0142159x.2014.874552
14. Wikipedia.https://en.wikipedia.org/wiki/Wikipedia:Size_comparisons#:~:text=Currently%2C%20the%20English%20Wikipedia%20alone,million%20articles%20in%20309%20languages.
15. Ranard, B. L., Ha, Y. P., Meisel, Z. F., Asch, D. A., Hill, S. S., Becker, L. B., . . . Merchant, R. M. (2014). Crowdsourcing—harnessing the masses to advance health and medicine, a systematic review. *Journal of general internal medicine, 29*(1), 187-203.
16. van der Heijden, H. (2004). User acceptance of hedonic information systems. *Mis Quarterly, 28*(4), 695-704.

**Figures**

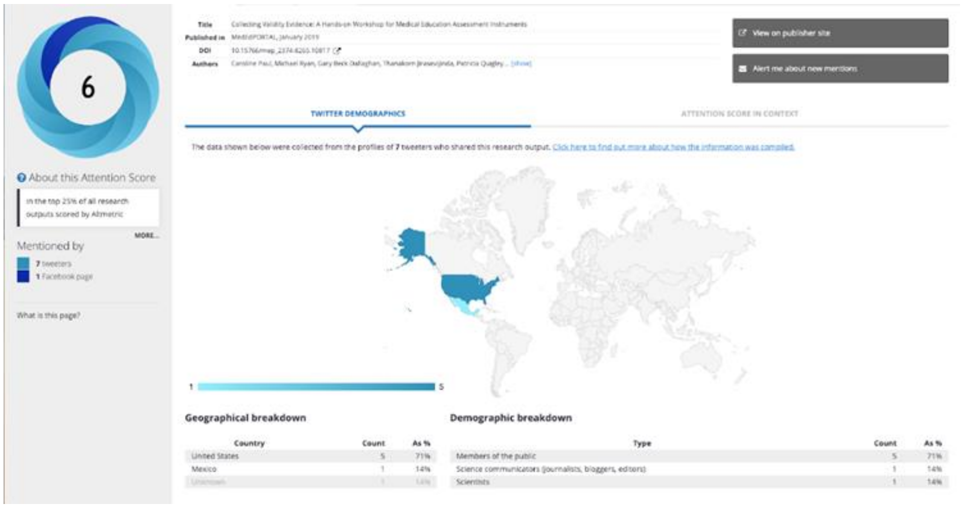


**Fig. 1.** The MedPortal page for social media and the downloading of geographic information

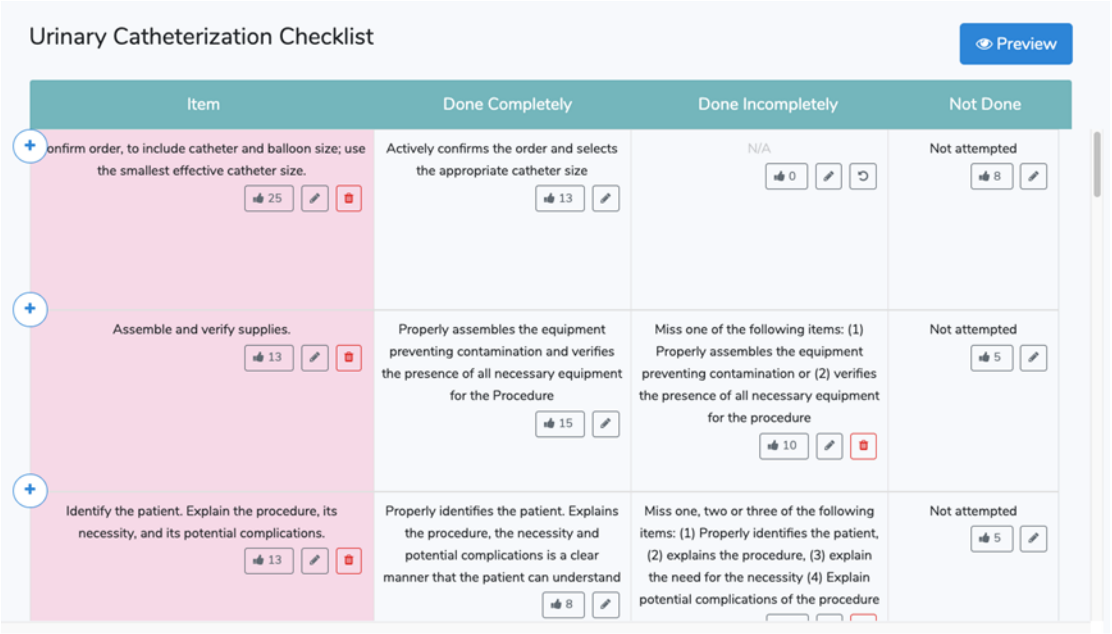


**Fig. 2.** The CAAT (beta 1.0) front page of the system displaying procedure-based OSCE checklists and online platforms.

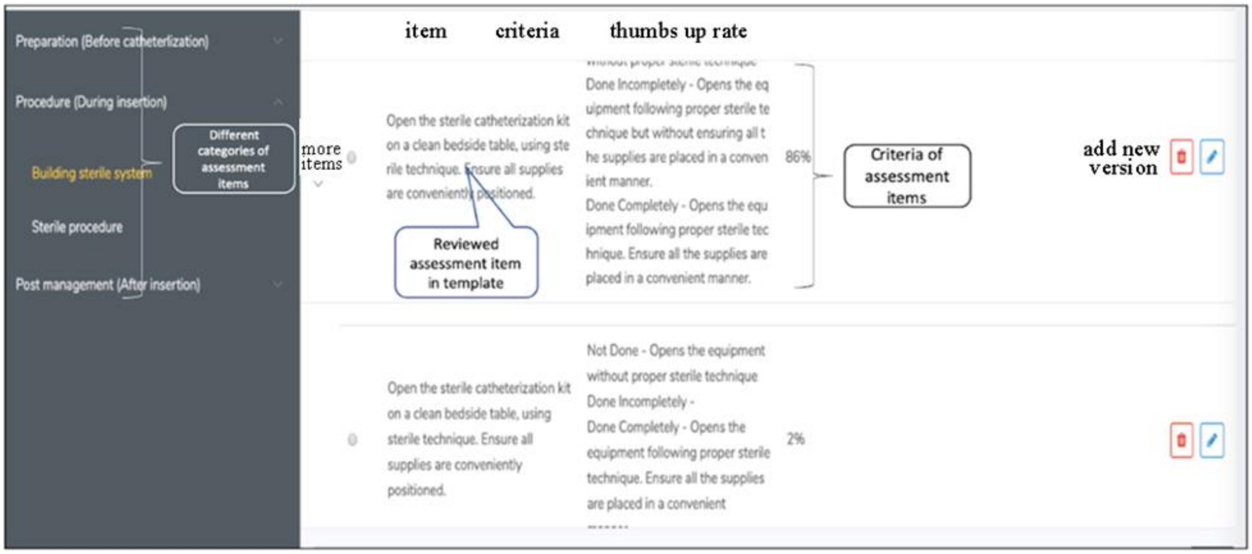


**Fig. 3.** The front page of the CAAT system version 1.0.

## Tables

**Table 1**. Demographics and characteristics of participants

| Characteristics | Statistics |
|---|---|
| Gender | |
| Male | 34 (68%) |
| Female | 16 (32%) |
| Area | |
| English | 15(30%) |
| Non-English | 35 (70%) |
| Occupation | |
| Physician | 41 (82%) |
| Other | 9 (18%) |
| Teaching experience (years) [a] | 8 (5 to 12.5) |
| Experience about checklist (years) [a] | |
| Using checklists | 6 (3 to 10) |
| Developing checklists | 4.5 (2 to 10) |

| Time of building checklist (minute) [a] | |
|---|---|
| Traditional method | 90 (45 to 120) |
| CAAT | 20 (10 to 40) |

**Table 2.** The overall CAAT acceptance (test value: 5)

| Item | MD | 95% CI | | *t* | Cohen's | *Post hoc* | *p* |
|---|---|---|---|---|---|---|---|
| | | Lower | Upper | | *d* | *power* | |
| 01 | .94 | .66 | 1.22 | 6.800 | 0.93 | 0.94 | < .001 |
| 02 | 1.00 | .76 | 1.24 | 8.250 | 1.15 | 0.99 | < .001 |
| 03 | .92 | .65 | 1.19 | 6.890 | 0.94 | 0.95 | < .001 |
| 04 | .84 | .51 | 1.17 | 5.090 | 0.71 | 0.80 | < .001 |
| 05 | 1.06 | .81 | 1.31 | 8.421 | 1.18 | 0.99 | < .001 |
| 06 | 1.22 | .96 | 1.48 | 9.480 | 1.30 | 0.99 | < .001 |
| 07 | 1.16 | .95 | 1.37 | 11.108 | 1.53 | 0.99 | < .001 |
| 08 | 1.02 | .74 | 1.30 | 7.366 | 1.01 | 0.97 | < .001 |
| 09 | 1.02 | .77 | 1.27 | 8.087 | 1.13 | 0.99 | < .001 |
| 10 | .82 | .51 | 1.13 | 5.358 | 0.73 | 0.82 | < .001 |
| 11 | .80 | .52 | 1.08 | 5.838 | 0.79 | 0.87 | < .001 |
| 12 | .76 | .48 | 1.04 | 5.367 | 0.75 | 0.83 | < .001 |
| 13 | 1.08 | .79 | 1.37 | 7.584 | 1.03 | 0.97 | < .001 |
| 14 | .88 | .53 | 1.23 | 5.088 | 0.70 | 0.79 | < .001 |
| Useful | .93 | .70 | 1.15 | 8.203 | 1.15 | 0.99 | < .001 |
| Ease | 1.12 | .91 | 1.32 | 10.792 | 1.51 | 0.99 | < .001 |
| Enjoyable | .85 | .59 | 1.11 | 6.611 | 0.91 | 0.94 | < .001 |
| BI | .98 | .69 | 1.27 | 6.861 | 0.94 | 0.95 | < .001 |

One-sample *t*-test; BI, behavior intention; CI, confidence interval; MD., mean difference

**Table 3.** Difference in developing a checklist between the CAAT and traditional method.

| Group | CAAT | | Tradition | | *t* | Cohen's | *Post hoc* | *p* |
|---|---|---|---|---|---|---|---|---|
| | M | SD | M | SD | | *d* | *power* | |
| **Overall** | 65.76 | 205.92 | 167.90 | 501.25 | -2.36 | -0.61 | 0.68 | .022 |
| **Language**[a] | | | | | | | | .010 |
| English | 25.20 | 20.25 | 54.67 | 36.57 | -2.98 | -1.59 | 0.88 | |
| Non-English | 83.14 | 244.75 | 216.43 | 594.54 | -2.18 | -0.75 | 0.69 | .036 |
| **Experience for checklist development**[b] | | | | | | | | |
| Junior | 41.12 | 70.80 | 96.60 | 75.44 | -3.76 | -1.54 | 0.98 | .001 |
| Senior | 90.40 | 283.36 | 239.20 | 704.77 | -1.75 | -0.71 | 0.52 | .093 |

Paired *t*-test. M, mean; SD, standard deviation. [a] English speaker (n = 15) and non-English speaker (n = 35); [b] senior (experience on developing checklist more than 4 years, n = 25) and junior (experience on developing checklist within 4 years, n = 25).
