# Supplementary material for: Crowdsource authoring as a tool for enhancing the quality of competency assessments in healthcare professions
Source: PLoS One. 2023 Nov 2;18(11):e0278571. doi: 10.1371/journal.pone.0278571 (PMC10621860; doi:10.1371/journal.pone.0278571)
Supplement: Attachment — Submitted filename: response sheet.docx [file pone.0278571.s010.docx]

response sheet

| Reviewer 1 | Provide the demographic characteristics of phases 1 and 2 participants. | The demographics for Phase I and II participants are found on lines 97, 116 – 119 (red highlights. For the study participants, the demographics are found in Table 1. |
| --- | --- | --- |
|  | Authors should report the results for each phase of study separately. So, they should re-organize the results section and provide the findings of all study phases. | We reported Phase I, II and the Study Phase results on lines 182 to 201 (please see the red highlights) |
|  | In discussion, authors should compare the performance of designed tool with the same ones to highlight the strengths of their work. | The comparisons are found on lines 256 – 279: The average time for participating experts was 65.76 minutes when using CAAT, whereas, they spent 167.90 minutes using OSCE, the traditional method currently used worldwide [1-3]. According to the study’s results, the average time saved by using CAAT is 102.14 minutes (Table 3). It showed that the CAAT system really saves the time of checklist design. The template function can guide the checklist designer the basic and important assessment criteria of particular skill. Moreover, the editing function also provides the individualized checklist for particular skills. The CAAT provides an efficient and innovative way to design the checklist. |
| Revier 2 | I was unable to see when the study took place. | The study occurred between October 2018 and May 2019 (we rewrote it in the abstract). It is also found in the methods section lines 83 and 86 (red highlights). |
|  | In the discussion it would be interesting to hear more about how the tool could be developed. | In the discussion we have added more about the tool on lines 284-294:  In the crowdsourcing methodology, the quality of the template is a critical issue. The user will modify the template and create a checklist to fit their particular settings. If the quality of the template is inadequate the users may spend a significant amount of time editing and improving the content. It is necessary to implement some mechanism to ensure the content of the template meets certain quality metrics. Currently, we use experts to review the uploaded checklist template. In the future, Natural Language Processing (NLP) based Artificial Intelligence (AI) may help determine if templates meet quality standards and suggest modifications to improve checklist generalizability.  In future developments, we will encourage the OSCE experts to upload more checklist templates. We will provide the checklist bank to the scholars who will design the checklists. The more faculties use and edit the checklists the more accurate and efficient the template will be. Moreover, extended use AI in the future will help to review the templates. |
