# Supplementary material for: Crowdsource authoring as a tool for enhancing the quality of competency assessments in healthcare professions
Source: PLoS One. 2023 Nov 2;18(11):e0278571. doi: 10.1371/journal.pone.0278571 (PMC10621860; doi:10.1371/journal.pone.0278571)
Supplement: Attachment — Submitted filename: Response to reviewers_CAAT.docx [file pone.0278571.s011.docx]

Response to reviewers

Reviewer 2: From my initial review..."The manuscript was generally very positive in tone and could at times have been more critical for example, what are some of the disadvantages of Wikipedia and how could these be address with this new methodology?" Could the introduction be reviewed with a more critical eye?

Response: We thank the reviewer for raising this important issue and provide the following addition to our manuscript along with two new references as support. This is found in our revised manuscript on lines 77 to 87, 303 to 316, 391, 392, and 395 to 397:

Crowdsourcing has its downsides and limitations. The frequently discussed ones include confidentiality, misleading, and intellectual property rights (IPR) issues. Crowdsourcing is a practice to update or add to the information available online by soliciting the contribution of the general public to accomplish a task. All the information is public, therefore, confidentiality is a given limitation of crowdsourcing. A crowdsourced task is done by masses of people, and one of the risks is misleading. Due to a lack of knowledge or bias, the crowd might take the project in the wrong direction. Also, since all the content is from the crowd, IPR has always been a debate. Further, there is concern about collective wisdom theft since the information is publicly available (Gao et al, 2011). Once the CAAT is built it must be tested to demonstrate usefulness and efficiency and also understand how to eliminate/resolve the possible issues mentioned.

CAAT adapted some of Wikipedia’s while also verifying the user’s identity. The user’s instructor identity needs to be confirmed before being allowed into the system. The user is also required to sign the NDA (non-disclosure agreement). These mechanisms will be able to overcome some of the shortcomings of crowdsourcing. The content on CAAT is all about assessment checklists. Therefore, it is important to ensure that students have no access to the content. During the onboarding process, the user’s competence in checklist design will reveal reducing the possibility of editing errors. Further, there are checklist templates available on CAAT. Templates are created and provided by skilled experts. Users can edit based on the template to address the specific needs of their institutions. The system allows users to edit within a direction range to reduce errors due to bias or lack of knowledge. When experts are solicited to provide templates, they need to sign an IPR (intellectual property rights) release to the system. At login, users are reminded that their editing will be logged by the system, and the IPR belongs to CAAT. The NDA ensures that the checklists will not leak. These are the mechanisms to help CAAT enjoy the benefits of crowdsourcing and avoid its downsides.

Gao, H., Barbier, G., & Goolsby, R. (2011) Harnessing the Crowdsourcing Power of Social Media for Disaster Relief. IEEE, 1541-1672/11/

Doan, A., Ramakrishnan, R., & Halevy, A. Y. (2011). The practice of crowdsourcing is transforming the Web and giving rise to a new field. Communications of the ACM, 54(4), 86-96
